# Enhancing Data Collection Through Optimization of Laser Line Triangulation Sensor Settings and Positioning

**DOI:** 10.3390/s25061772

**Published:** 2025-03-12

**Authors:** Dominik Heczko, Jakub Chlebek, Jakub Mlotek, Tomáš Kot, Lorenzo Scalera, Martin Dekan, Zdeněk Zeman, Zdenko Bobovský

**Affiliations:** 1 Department of Robotics, Faculty of Mechanical Engineering, VSB–Technical University of Ostrava, 70800 Ostrava, Czech Republic; jakub.chlebek@vsb.cz (J.C.); jakub.mlotek@vsb.cz (J.M.); tomas.kot@vsb.cz (T.K.); zdenek.zeman@vsb.cz (Z.Z.); zdenko.bobovsky@vsb.cz (Z.B.); 2 Polytechnic Department of Engineering and Architecture, University of Udine, 33100 Udine, Italy; lorenzo.scalera@uniud.it; 3 Institute of Robotics and Cybernetics, Slovak University of Technology in Bratislava, 812 19 Bratislava, Slovakia; martin.dekan@stuba.sk

**Keywords:** laser scanner, sensor placement, reliability, triangulation, in plane, out of plane

## Abstract

This study proposes a new approach to improving laser sensor data collection through optimised sensor settings. Specifically, it examines the influence of laser sensor configurations on laser scanning measurements obtained by using a laser line triangulation sensor for transparent and non-transparent plastics, as well as aluminium alloys. Distance data were acquired with a three-degree-of-freedom positioning device and the laser sensor under both manual and automatic settings. Measurements were performed at the sensor’s reference distance and across a wide range of positional configurations. The results of extensive experimental tests highlight optimal sensor configurations for various materials and sensor orientations relative to the scanned surface, including both in-plane and out-of-plane angles, to enhance the reliability and accuracy of distance data collection.

## 1. Introduction

Laser and optical sensors represent a key technology in modern industry, as well as in non-industrial areas, for high-precision distance measurement. Outside of industry, laser sensors are applied in medicine, for example, in measuring the thickness of the cornea during ophthalmological examinations [1]. They are also used in the creation of artificial organs or joints by using additive technology, where laser sensors scan parts of the human body and subsequently create a model of the organ [2].

In geodesy, laser sensors enable accurate terrain mapping, distance measurement, slope measurement, and height differences with high precision and speed. They serve to digitise terrain models or monitor terrain deformations [3,4]. Additionally, laser sensors are essential to the creation of 3D models for virtual reality, the gaming industry, and realistic reconstruction of buildings [5]. However, with increasing popularity and improved artificial intelligence, the platforms being developed for these purposes are capable of generating structured and unstructured environments [6,7].

Laser sensors have already become a part of agricultural systems, often integrated into mobile robots in modern orchards. These devices monitor and collect data on plants and their fruits, particularly their geometric characteristics [8]. The information obtained provides farmers with important data to plan and manage key operations, such as irrigation, fertilisation, plant pruning, and prevention of disease spread to other plants [9].

In industry, laser scanning is widely used for various applications. These include object localisation for bin-picking applications [10], product inspection, and accurate high-speed dimensional measurement [11]. It is also essential to reverse engineering, where detailed digital models of existing objects are created for analysis and modifications [12]. Additionally, laser scanning is used to monitor manufacturing processes, such as the application of adhesive or sealant layers; inspect welds [13]; and ensure adherence to technological procedures. Another important use is in additive manufacturing, where it is used to monitor the application of material layers [14,15] and check the general condition of an object, including quality control during printing [16].

Laser sensors enable contactless measurement, preventing sensor wear and avoiding damage to the measured object. They provide high precision and, most importantly, fast measurement, which is crucial to meeting the increasingly demanding conditions of production line cycles. Another key advantage is their ability to measure distances or other control variables even in hard-to-reach areas, which is often problematic for contact sensors. Laser sensors also enable the creation of large point clouds, aiding in the digitisation of undocumented spaces for subsequent analysis [17] or the digitisation of unstructured environments [18].

**Figure 1:**
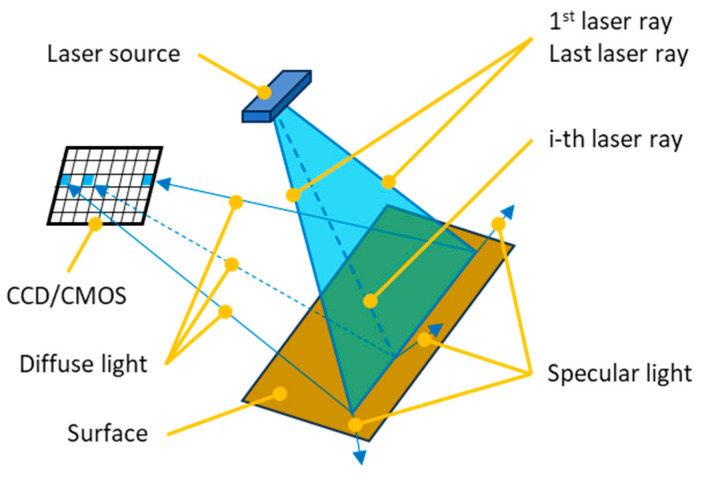
The principle of triangulation on a 2D line sensor.

In industrial environments, triangulation laser sensors (1D, 2D, or 3D) are often preferred over contact sensors for several reasons. These sensors operate on the principle of triangulation [19]. They project a laser beam towards the target and capture the reflected beam by using light-sensitive chips, such as a Complementary Metal-Oxide-Semiconductors (CMOSs) or Charge-Coupled Devices (CCDs). The angle at which the reflected beam is captured is then analysed to calculate the distance from the target; see Figure 1.

However, laser triangulation sensors have certain limitations that need to be considered. One of the main constraints is the potential for laser interference from external factors, such as ambient light sources or reflections [20]. These interference phenomena can lead to measurement errors or even complete sensor failure. For instance, a red laser cannot be used to scan red-hot steel because the measured object emits light at the same or similar wavelength (625 to 740 nm). This limitation can be overcome by using a laser with a different wavelength; blue lasers with a wavelength of 405 nm are often used [21]. Another limitation could be an operating environment where heavy dust, fog, or smoke is present. Under these harsh conditions, laser sensors may be unusable due to reduced transmission of the laser beam. The geometry of the measured object also poses a challenge. Because of the principle of triangulation, the incomplete scanning of the object can occur, leading to so-called blind spots.

One of the main limitations is the sensor’s sensitivity to surfaces with varying reflectivity. When scanning objects with different materials or surface finishes, incorrect measurements or insufficient data recording can occur. However, this problem is partially mitigated by High Dynamic Range (HDR), which allows for the automatic adjustment of the laser beam power depending on the surface reflectivity [22].

**Figure 2:**
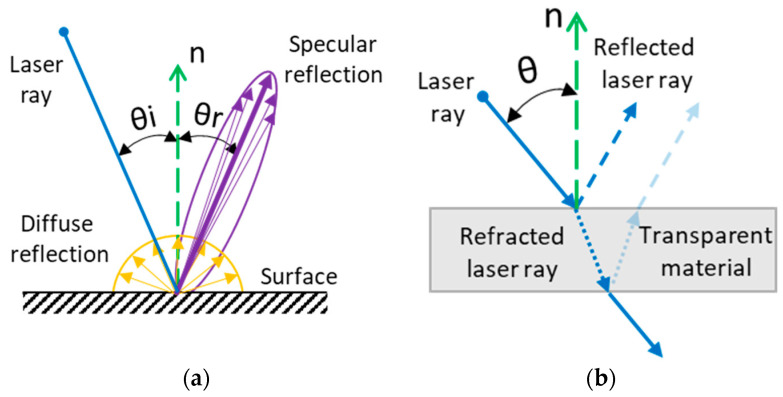
Undesirable effects in laser scanning: (**a**) specular reflection, where n is the surface normal, θ_i_ is the angle of incidence, and θ_r_ is the angle of reflection; (**b**) refraction of the laser beam, where n is the surface normal and θ is the angle of incidence of the beam.

Reflectivity is the ability of a material to reflect light, which depends on its optical properties. There are two main types of light beam reflection: diffuse and specular reflection [23], as shown in Figure 2a. Both types of reflection can exist simultaneously, but one usually dominates. In laser scanning, diffuse reflection is the preferred type. A surface with a high light-scattering index is called a Lambertian surface [24]. Specular reflection is dominant in shiny and exceptionally smooth materials, whereas matte materials are characterised by the predominance of diffuse reflection.

Beyond these traditional types of reflection, it is worth to mention metasurfaces—engineered two-dimensional structures capable of manipulating light in unprecedented ways. By controlling the phase, amplitude, and polarisation of light at the subwavelength scale, metasurfaces enable phenomena like anomalous reflection, where the reflection angle can deviate from the conventional laws of reflection. This effect has been demonstrated in all-dielectric gradient metasurfaces for visible light [25] and in broadband metasurfaces optimised for efficient reflection [26]. These capabilities offer new possibilities for advanced optical device design and precise control over light propagation.

Digitising almost mirror-like and highly reflective surfaces continues to be a challenge, despite significant progress in this field. The emitted light reflects off these surfaces in a mirror-like manner, often away from the CCD/CMOS chip, making it undetectable to the sensor. However, it is possible to scan these surfaces under certain conditions, as demonstrated in a study [27].

When light interacts with transparent materials, it also encounters the effects of light refraction. Upon striking a transparent surface, a portion of the light beam is absorbed, while the rest splits; some reflects off the surface, and the remainder travels through the transparent medium, such as water or glass, as depicted in Figure 2b. This phenomenon results in the detection of multiple reflections, typically from the top and bottom surfaces. However, this effect can be used to measure the thickness of the material or is mitigated by using masks or employing a special light spectrum [28].

In general, these phenomena can be minimised by applying a layer of matte material, which dulls the surface and improves diffuse light scattering into the surroundings. However, this approach may not always be feasible, especially in the automotive industry, where the application of materials to visible parts such as headlights is prohibited. Therefore, it is important to address this issue.

**Figure 3:**
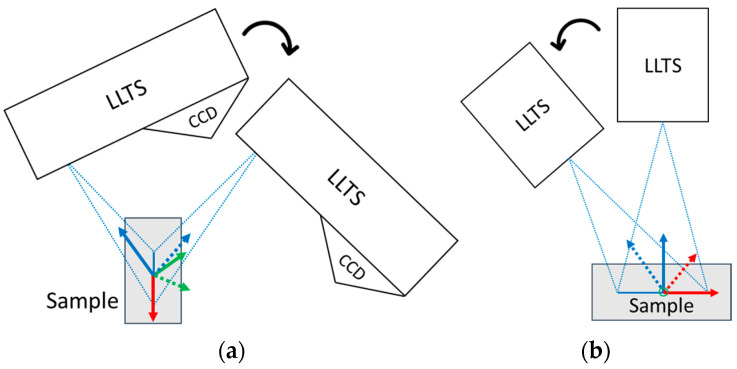
Positioning of laser line triangulation (LLT) sensor. The colored arrows indicate the sensor’s reference frame: red represents the X-axis, green represents the Y-axis, and blue represents the Z-axis. Dashed reference frame indicating orientation at a given angle: (**a**) out-of-plane angles, representing rotation around the *X*-axis; (**b**) in-plane angles, representing rotation around the *Y*-axis.

In our previous research study [27], we examined the effect of the angle of incidence (AoI) of a laser beam on its reflected intensity and proposed recommendations for sensor positioning relative to the object to improve the reliability of data collection. These recommendations, along with the properties of different materials, were tested in the study [29], which included a comparison of virtual and real scanning. This approach was suitable for flat surfaces, assuming that the sensor was placed perpendicular to the surface or at an out-of-plane angle (refer to Figure 3a). It was primarily used to measure geometric attributes such as radius, angles, gaps, and ovality. However, these characteristics are no longer valid when measuring at in-plane angles (see Figure 3b) or in combination with out-of-plane angle. This is especially relevant for the comprehensive scanning of entire objects to generate a detailed point cloud, where the sensor positioning must be adjusted or the component is highly geometrically complex.

It should be noted that sensor orientation has an impact on the quality of the resulting point cloud [30]. However, systematic and random errors resulting from positioning at in-plane and out-of-plane angles can be eliminated by using the LLT sensor error model, as presented in [31]. Furthermore, in our previous research study [27], we focused on ensuring reliable data acquisition with laser triangulation scanners by analysing the intensity of the reflected laser. The optimal scanning position was identified as the one that yields the highest possible intensity, excluding areas affected by specular reflection. However, the reliance on the reflected intensity posed limitations due to the sensor’s saturation threshold. When this threshold is exceeded, the sensor registers a maximum intensity value, even though the actual intensity might be significantly higher, resulting in data loss—a phenomenon analogous to overexposure in photography. To address these limitations, we have changed our approach in the current study. Instead of relying on the reflected intensity, we evaluate the number of detected points in various scanning positions and their associated accuracy. This approach provides a more robust framework for analysing data reliability across different materials and surface conditions.

In this article, we emphasise the importance of the sensor’s positioning in relation to the scanned surface to ensure reliable data collection. Specifically, we highlight how the sensor’s parameters and its orientation relative to the scanned object influence both the number of detected points and the accuracy of the measurements. The main contributions of this work lie in presenting a systematic evaluation of these effects and providing insights into optimal sensor configurations for both common and challenging materials. Moreover, our approach offers significant practical advantages for industrial applications. By identifying the optimal positioning of the LLT sensor to ensure reliable data collection, the design and deployment of scanning workstations can be accelerated while reducing costs. Knowing the ideal sensor configuration eliminates the need for extensive testing and experimentation with different hardware setups. A key advantage of our method is that it works purely with the sensor itself, without requiring additional investments in new hardware or software. By positioning and orienting the sensor correctly and setting the right parameters, users can achieve more accurate and reliable measurement results. Existing workstations can also be adjusted to achieve even better measurement performance, enhancing their efficiency and data quality.

## 2. Methodology

In our previous research study [27], a UR10e robot was used for sensor positioning to collect data, which was sufficient for positioning at out-of-plane angles. However, due to its kinematic structure, dynamic effects, and the construction of its joints, vibrations occurred after the robot stopped. This required a longer waiting period for the system to stabilise when changing positions. Additionally, as the joints heated up, the positioning accuracy decreased, as noted in [32]. Although temperature effects could be compensated for, as also demonstrated in [32], this compensation would be significantly time-consuming for complex motions. To address these challenges and improve movement variability, a three-degree-of-freedom positioning device was developed.

**Figure 4:**
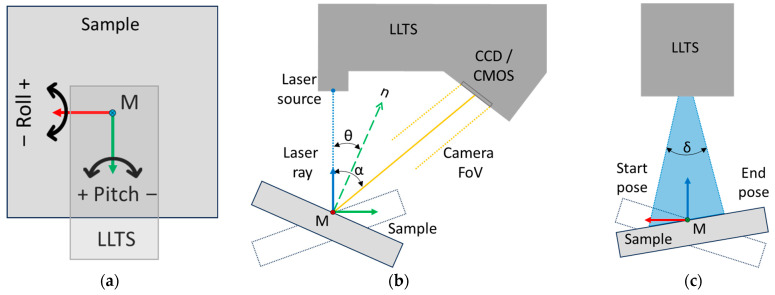
Data acquisition scheme from experimental measurements. The colored arrows indicate the sensor’s reference frame: red represents the X-axis, green represents the Y-axis, and blue represents the Z-axis.: (**a**) top view of the sensor, illustrating the tilting of the sample around the X- and Y-axes; (**b**) side view of the sensor, illustrating the tilting around the *X*-axis and the basic parameters of the sensor, where θ is the AoI of the laser beam relative to the surface normal n, and α is the triangulation angle; (**c**) front view of the sensor, illustrating the tilting around the *Y*-axis, where δ is the angle of the laser projection cone.

The positioning device allows the sample to tilt around the *X*-axis (roll angle) and the *Y*-axis (pitch angle) and move linearly along the *Z*-axis, as illustrated in Figure 4a, which represents a top view of the sensor. The sample rotates around point M, which is defined by the intersection of the X- and Y-axes. For data collection, the sample was gradually tilted around the *X*-axis from −70° to +40°, as indicated in Figure 4b, which represents a side view of the sensor. Subsequently, the sample was tilted by a specified step around the *Y*-axis, and the measurements were repeated around the *X*-axis. This process was repeated until measurements were taken at all positions around the *Y*-axis from −40° to +45°, as shown in Figure 4c, which represents a front view of the sensor. The step between positions was 1°; therefore, the data for one sample were collected from a total of 9546 positions.

## 3. Experiment Setup and Data Collection

**Figure 5:**
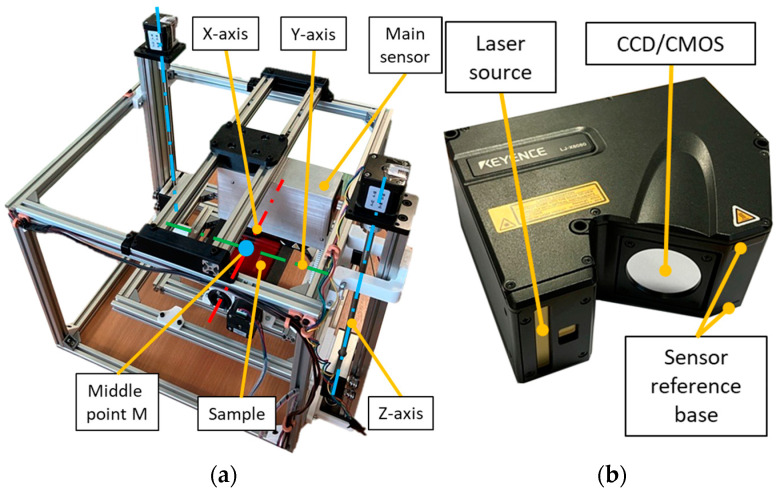
Experimental setup: (**a**) positioning device with three degrees of freedom; (**b**) Keyence LLT LJ-X8080 sensor in detail.

The positioning device (shown at Figure 5a) allowed the samples to tilt at two angles —roll and pitch—and allowed for the adjustment of the sample’s distance from the sensor. For measurements, we used an LLT sensor LJ-X8080 [33], manufactured by Keyence. This sensor features a blue laser with a wavelength of 405 nm, which prevents interference from ambient light. Additional sensor parameters are provided in Table 1.

### 3.1. Experimental Workplace

The positioning device was assembled from aluminium profiles and standard structural components, with custom-designed parts produced through additive manufacturing. These parts have been modified to improve their resistance to temperature, as described in [34]. The total weight of the system, including the sensor (1.1 kg), was 10.5 kg. The individual axes were driven by stepper motors. To calibrate the positioning device, two LLT sensors were used to measure the tilt in each axis, using the manufacturer’s software (Terminal Software v 1.2.0). This calibration ensured that the platform holding the sample was set to a default position perpendicular to the projected laser beam. The measuring station is shown in Figure 5a. The *Z*-axis was adjusted for each sample according to its thickness. The sensor measures the distance of individual points relative to the base of the sensor, which is shown in Figure 5b.

**Figure 6:**
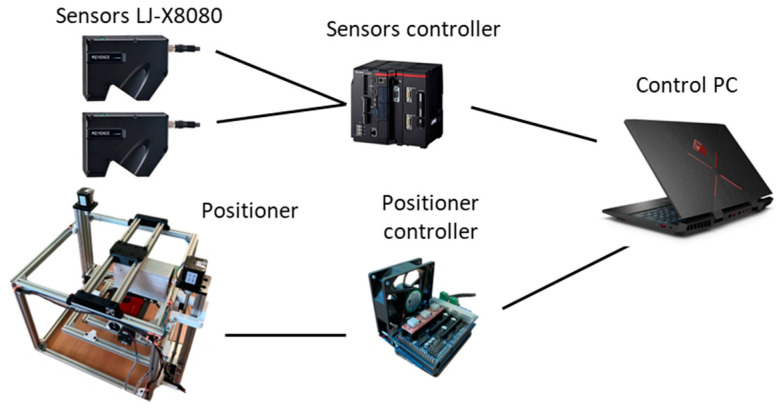
A schematic diagram of the workstation.

The positioning device was controlled through a custom-developed application running on the control computer. This application, written in C#, provided a user interface and managed real-time command execution and data acquisition from the device. The positioning device was connected to the control computer via USB. When a command was sent to the positioning device, the control unit provided feedback once the movement was completed. Subsequently, a command was sent to the sensor control unit to initiate data collection. The sensor control unit communicated with the computer through Ethernet. The connection diagram is shown in Figure 6.

The measured samples had varying dimensions and surface roughness, as shown in Table 2. In the zero position, with the roll and pitch angles set to 0°, the sensor was placed at distance of 73 mm from each sample. This reference distance was specified by the sensor manufacturer and represents the midpoint of the sensor’s measurement range along the *Z*-axis.

**Figure 7:**
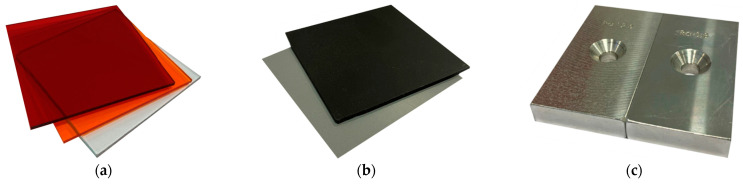
Measured samples: (**a**) transparent plastics; (**b**) non-transparent plastics; (**c**) aluminium alloys with different roughness (face milling, Ra 0.8–12.5).

The samples chosen for this experiment are commonly used materials in the automotive industry. These samples include transparent acrylic plastics in red, orange, and clear colours (Figure 7a), black and grey plastic sheets (Figure 7b), and aluminium alloy samples with different levels of roughness after face milling (Figure 7c).

### 3.2. Data Collection

After the sample was inserted, the positioning device was calibrated with the aid of two LLT sensors, which measured the sample’s tilt and ensured precise alignment of the sample in the positioning device. The main LLT sensor (as seen in Figure 5a) measured the tilt of the platform around the *X*-axis and the distance from the sample along the *Z*-axis. The auxiliary LLT sensor measured the tilt around the *Y*-axis. Following calibration, the auxiliary sensor was removed to enable unrestricted movement of the positioning device during measurement. The main sensor projected a laser line onto the sample, aligned with the axis of rotation around the *X*-axis. The rotation around the *Y*-axis was perpendicular to the *X*-axis and passed through the midpoint of the X-range, corresponding to the central point M of the positioning device, as illustrated in Figure 5a.

The measurement sequence began by setting the sample’s tilt angle around the *Y*-axis within a range of −40° to +45°. Subsequently, the tilt angles around the *X*-axis were adjusted within a range of −70° to +40°. At each position, a waiting period was allotted for the sample to stabilise after completing its movement. After stabilisation, profile data (points) were collected for further processing. This procedure was repeated until all positions were scanned, resulting in a complete dataset. The measurement process is detailed in Algorithm 1.
**Algorithm 1.** The measurement process.**for** i = −40 to 45    //i represents the pitch angle (rotation around the *Y*-axis)    SetPitchPose (i)    **for** j = −70 to 40    //represents the roll angle (rotation around the *X*-axis)        SetRollPose (j)        WaitForStabilization ()        CollectProfileData ()    **end for** **end for**

Some sensors require warming up to their operating temperature to ensure accurate measurements. Although this sensor does not require preheating, it was allowed to warm up prior to data collection. For this sensor, the operational temperature did not affect the number of captured points. However, it did influence the accuracy of the entire profile. The accuracy was measured at a single position with multiple scans, immediately after the sensor was powered on, and subsequently at 30 min intervals up to 90 min. After 90 min of operation, a deviation of ±0.016 mm was observed when comparing scans taken at room temperature and after the warm-up period. While this level of deviation may be negligible for some applications, it could become critical in scenarios that demand high precision.

Data were collected by using both manual and automatic sensor settings, with individual parameters configured before each measurement, adjusted to the specific sample and measurement type. In manual mode, the parameters were set based on the user’s observation and estimation. In automatic mode, the sensor applied the “Sensitivity adjustment” function to determine the optimal settings. An overview of the configurable parameters and their possible values is provided in Table 3, based on the sensor datasheet. The specific parameter values used for each sample and type of measurement are listed in Table 4. When using automatic adjustment, the laser power was modified during measurements in response to the reflected beam intensity.

## 4. Experimental Results

All samples used in this experiment were flat, resulting in an expected point profile that should form a straight line. However, due to overexposure, noise, and material properties, scanning inaccuracies can occur. These inaccuracies cause some points to deviate significantly along the *Z*-axis, resulting in a noisy profile. To evaluate the accuracy of the scanned profiles, we employed linear regression, as the expected profile should be linear.

**Figure 8:**
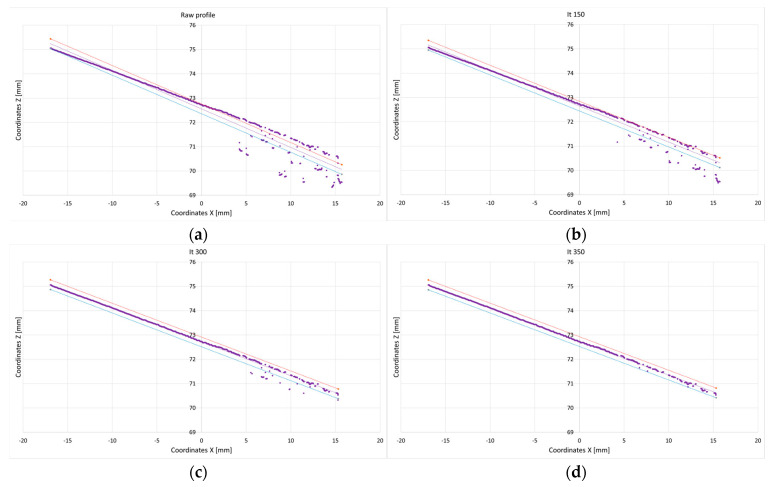
Iterative accuracy calculation procedure. The profile data (points) are marked with a bold purple, the regression line is indicated with a purple dotted line, the positive threshold (+0.05 mm) is represented by a red dashed line, and the negative threshold (−0.05 mm) is shown as a blue dotted line: (**a**) raw profile; (**b**) iteration No. 150; (**c**) iteration No. 300; (**d**) iteration No. 350.

An iterative approach was used to refine the profile. In each iteration, the most distant point outside the defined accuracy threshold was removed. The regression equation was then recalculated, and the profile was reevaluated in subsequent iterations without the removed points. The accuracy threshold was set to 0.1 mm (±0.05 mm) along the Z-axis of the regression line. The final accuracy of the profile was determined by the number of points that remained within this threshold. The iterative procedure to calculate the accuracy of the profile is shown in Figure 8a–c.

The data for each sample are visualised by using two heat maps, where each pixel represents a discrete position in which the measurement (scan) was performed, for a total of 9546 positions.

**Figure 9:**
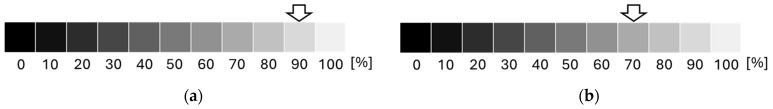
The determination of the grey level of a pixel in the heat map: (**a**) the number of points in the profile; (**b**) the accuracy of the profile.

The first heat map illustrates the number of detected points. The pixel intensity is represented in shades of grey, where black indicates 0% of the maximum detectable points and nearly white represents 100%. For example, a measurement detecting 2915 points out of a maximum of 3200 points would correspond to a grey level reflecting approximately 91% (see Figure 9a).

The second heat map depicts the accuracy of the profile. Again, the intensity of pixels is expressed in shades of grey, but it represents the number of points in the profile that fall within the specified accuracy threshold. For instance, if 2184 out of 2915 points meet the accuracy criterion of ±0.05 mm, the intensity of pixels reflects this proportion (approximately 75%; see Figure 9b).

These heat maps provide a comprehensive visual comparison of scanner performance in terms of both data completeness and accuracy for various positions and material samples. The intensity scale is divided into 10% increments in both heat maps, as shown in Figure 9a,b. As demonstrated by this example, a profile with a large number of points does not necessarily guarantee reliable data.

### 4.1. Non-Transparent Plastics

**Figure 10:**
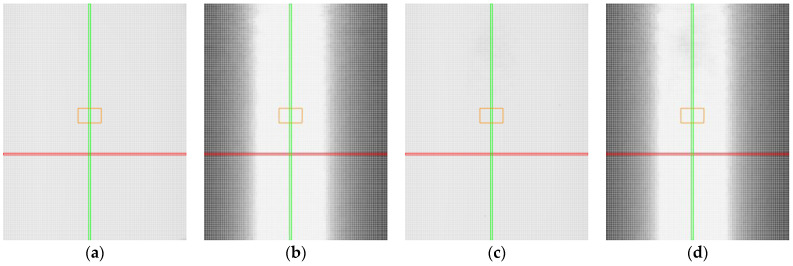
Heat maps for non-transparent black plastic. Red line represents position with roll value 0°, green line represents position with pitch value 0°, orange represents the area with mirror-like reflections: (**a**) captured points, manual settings; (**b**) accuracy of the profile, manual settings; (**c**) captured points, automatic settings; (**d**) accuracy of the profile, automatic settings.

Measurement results are presented by using heat maps, as shown in Figure 10, to illustrate the outcomes of different sensor settings. The red axis represents the roll with a tilt value of 0°, and the green axis corresponds to the pitch with a tilt value of 0°. The orange rectangle highlights the area with mirror-like reflections. The pixels in the heat maps represent individual positions where measurements were conducted, corresponding to specific combinations of roll and pitch angles.

At a first glance, the results obtained by using manual and automatic sensor settings for the non-transparent black plastic appear nearly identical. However, a closer analysis reveals notable differences. In certain areas, the automatic sensor settings resulted in a slight loss of detected points, as indicated by darker regions in the heat maps (Figure 10c). Furthermore, the accuracy of the profile was higher when the sensor was manually configured (Figure 10b). This is evident from the presence of grey pixels in the graphs corresponding to the automatic settings (Figure 10d), which indicate that a small number of points exceeded the specified accuracy threshold. The automatic settings dynamically adjusted the laser power, which may have resulted in data loss or reduced precision due to light absorption by the black surface.

**Figure 11:**
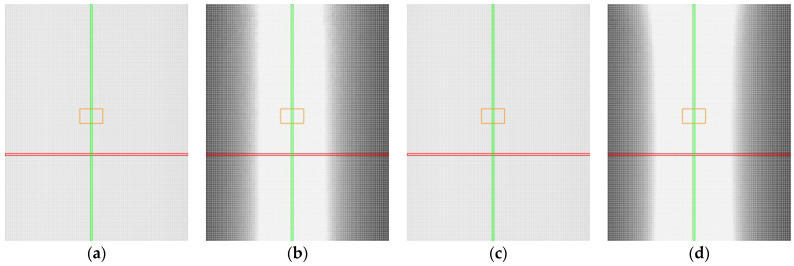
Heat maps for non-transparent grey plastic. Red line represents position with roll value 0°, green line represents position with pitch value 0°, orange represents the area with mirror-like reflections: (**a**) captured points, manual settings; (**b**) accuracy of the profile, manual settings; (**c**) captured points, automatic settings; (**d**) accuracy of the profile, automatic settings.

The heat maps for the non-transparent grey plastic demonstrate that both manual and automatic sensor settings performed similarly in terms of the number of captured points. Figure 11a,c show nearly identical results, with most positions reaching near-maximum detection levels, as indicated by the predominantly light shading across the maps. This consistency suggests that the material properties of the grey plastic did not significantly challenge the scanner’s ability to detect points, regardless of the sensor settings used.

However, in terms of accuracy, distinct differences can be observed between the two configurations. Manual settings (Figure 11b) yielded a large light-shaded area, indicating good accuracy across a significant portion of the measured positions. Notably, the automatic settings (Figure 11d) outperformed the manual settings, with an even larger light-shaded region, particularly in the upper part of the map. This indicates that the profiles measured under automatic settings were more precise in these positions.

### 4.2. Aluminium Alloys

Despite the varying surface roughness of the aluminium alloy samples, the resulting heat maps were very similar across all samples.

**Figure 12:**
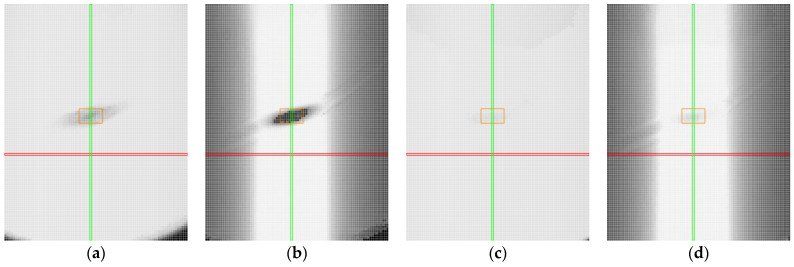
Heat maps for aluminium alloys with surface roughness Ra 0.8. Red line represents position with roll value 0°, green line represents position with pitch value 0°, orange represents the area with mirror-like reflections: (**a**) captured points, manual settings; (**b**) accuracy of the profile, manual settings; (**c**) captured points, automatic settings; (**d**) accuracy of the profile, automatic settings.

**Figure 13:**
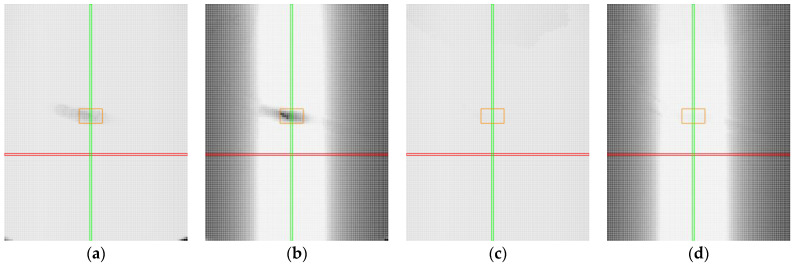
Heat maps for aluminium alloys with surface roughness Ra 1.6. Red line represents position with roll value 0°, green line represents position with pitch value 0°, orange represents the area with mirror-like reflections: (**a**) captured points, manual settings; (**b**) accuracy of the profile, manual settings; (**c**) captured points, automatic settings; (**d**) accuracy of the profile, automatic settings.

**Figure 14:**
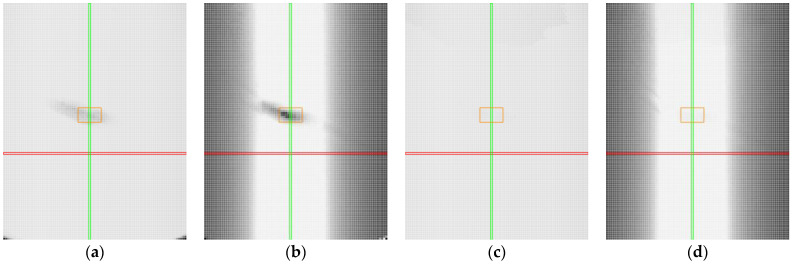
Heat maps for aluminium alloys with surface roughness Ra 3.2. Red line represents position with roll value 0°, green line represents position with pitch value 0°, orange represents the area with mirror-like reflections: (**a**) captured points, manual settings; (**b**) accuracy of the profile, manual settings; (**c**) captured points, automatic settings; (**d**) accuracy of the profile, automatic settings.

**Figure 15:**
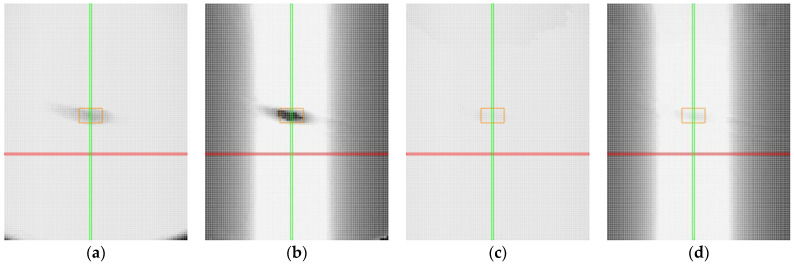
Heat maps for aluminium alloys with surface roughness Ra 6.3. Red line represents position with roll value 0°, green line represents position with pitch value 0°, orange represents the area with mirror-like reflections: (**a**) captured points, manual settings; (**b**) accuracy of the profile, manual settings; (**c**) captured points, automatic settings; (**d**) accuracy of the profile, automatic settings.

**Figure 16:**
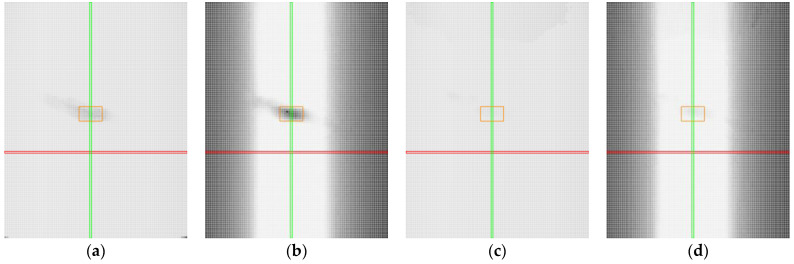
Heat maps for aluminium alloys with surface roughness Ra 12.5. Red line represents position with roll value 0°, green line represents position with pitch value 0°, orange represents the area with mirror-like reflections: (**a**) captured points, manual settings; (**b**) accuracy of the profile, manual settings; (**c**) captured points, automatic settings; (**d**) accuracy of the profile, automatic settings.

Regarding the number of points captured by using manual sensor settings, it was observed that fewer points were detected in the specular reflection region. This can be attributed to overexposure of the image, which caused data loss. Additionally, black spots indicating a low number of points were visible in the lower corners of the graphs, corresponding to extreme positive roll values (approximately 40°) and extreme pitch values (−40° and +45°), as shown in Figure 12a, Figure 13a, Figure 14a, Figure 15a and Figure 16a. For the sample with a surface roughness of Ra 0.8, this region was the largest, as seen in Figure 12a. In these positions, due to reduced sensor power, no light (reflected laser) returned to the sensor, resulting in minimal or no point detection. This was particularly evident for the Ra 0.8 sample, where the smooth surface caused the laser to reflect outside the sensor’s field of view. For samples with higher roughness, light was reflected to the sensor more effectively.

With automatic sensor settings, more points were generally captured, as illustrated in Figure 12c, Figure 13c, Figure 14c, Figure 15c and Figure 16c. Heat maps for all samples showed nearly 90% or more captured points. In the upper region of the graphs (roll of approximately −70° to −40°), the profile coverage was slightly lower, around 80% or more. However, in this region, manual settings detected more points (approximately 90%). In the specular reflection region, the profile coverage was also 80% or higher, while manual settings exhibited significant point loss in this region. For the Ra 0.8 sample, point loss occurred in the Roll 40° and Pitch 45° region (lower right corner), as shown in Figure 12c. This can be explained by the same phenomenon as observed with manual settings (a smooth surface causing the light to reflect away from the sensor).

In terms of accuracy, within the pitch range of ±17°, the accuracy was nearly 100%. However, with the increase in tilt toward the positive or negative extremes, the accuracy decreased sharply, as shown in Figure 12b,d, Figure 13b,d, Figure 14b,d, Figure 15b,d and Figure 16b,d. Notably, low accuracy was observed with manual settings in the specular reflection regions, as shown in Figure 12b, Figure 13b, Figure 14b, Figure 15b and Figure 16b. In contrast, the accuracy was significantly better with automatic settings. For samples with Ra 1.6 and Ra 3.2, the profile accuracy in the specular reflection region was consistent with the accuracy in the rest of the measured positions, as seen in Figure 13d and Figure 14d.

### 4.3. Transparent Plastics

**Figure 17:**
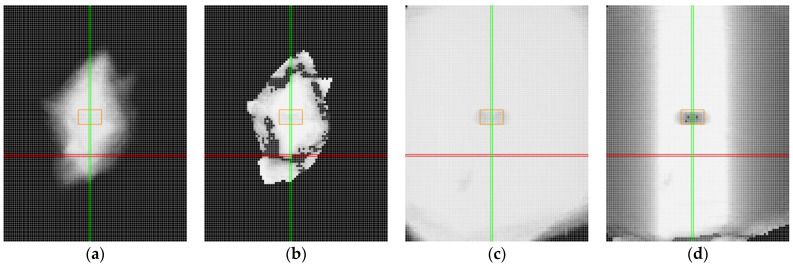
Heat maps for clear transparent plastic. Red line represents position with roll value 0°, green line represents position with pitch value 0°, orange represents the area with mirror-like reflections: (**a**) captured points, manual settings; (**b**) accuracy of the profile, manual settings; (**c**) captured points, automatic settings; (**d**) accuracy of the profile, automatic settings.

The differences between manual and automatic sensor settings were particularly pronounced when scanning the clear plastic sample. When using manual settings, the detected points were concentrated in a significantly smaller region, primarily around the area of specular reflections, as shown in Figure 17a. Outside this region, no points were detected, likely due to the fixed intensity of the laser, which could not adapt to the high transparency of the material. In contrast, automatic settings resulted in a much broader distribution of detected points, covering nearly the entire range of positions except for the extreme corners and the region of specular reflections, as indicated by darker pixels in Figure 17c.

In terms of accuracy, the manual settings exhibited relatively stable accuracy within the narrow vicinity of the specular reflection zone. However, further away from this zone, noticeable fluctuations in accuracy were observed, as shown in Figure 17b. These deviations are likely attributable to the properties of the material itself. Transparent plastics are not always perfectly homogeneous; internal defects or structural inconsistencies, invisible to the human eye, may refract or redirect the laser beam. This can lead to noise in the captured profile and reduce accuracy.

With automatic settings, the accuracy followed a trend similar to that observed with aluminium samples. Within the pitch range of ±17°, the accuracy was almost 100% but decreased rapidly with the increase in tilt angles in both the positive and negative directions. Additionally, the accuracy dropped at a roll angle of approximately 40°. A pronounced decrease in accuracy was also evident in the region of specular reflections, as shown in Figure 17d.

**Figure 18:**
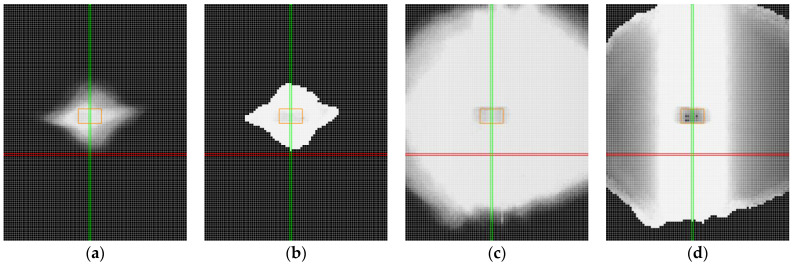
Heat maps for orange transparent plastic. Red line represents position with roll value 0°, green line represents position with pitch value 0°, orange represents the area with mirror-like reflections: (**a**) captured points, manual settings; (**b**) accuracy of the profile, manual settings; (**c**) captured points, automatic settings; (**d**) accuracy of the profile, automatic settings.

**Figure 19:**
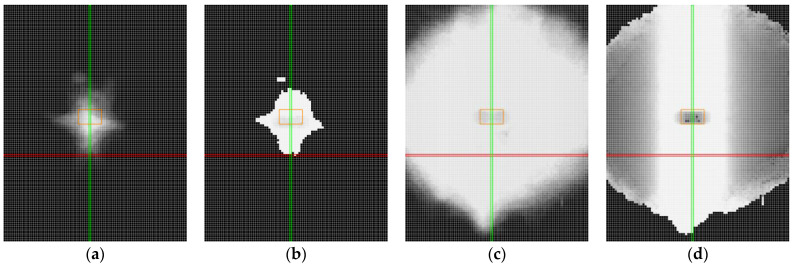
Heat maps for red transparent plastic. Red line represents position with roll value 0°, green line represents position with pitch value 0°, orange represents the area with mirror-like reflections: (**a**) captured points, manual settings; (**b**) accuracy of the profile, manual settings; (**c**) captured points, automatic settings; (**d**) accuracy of the profile, automatic settings.

For the red and orange transparent plastic samples, the number of detected points with manual sensor settings was very similar for both materials. Points were detected only around the specular reflection region, as shown in Figure 18a and Figure 19a. However, this detected area was slightly smaller than that observed with clear plastic. Automatic settings, while providing a larger detection range compared with manual settings (Figure 18c and Figure 19c), still showed a smaller area than that achieved with automatic settings for clear plastic. Additionally, in both materials, point loss was observed in the specular reflection region.

In terms of accuracy, the manual sensor settings demonstrated high precision (90–100%), as depicted in Figure 18b and Figure 19b. The accuracy achieved with the automatic settings was similar to that seen with the clear plastic sample. Within the pitch angle range of ±17°, the accuracy was close to 100% but dropped significantly with the increase in tilt angles in either direction. A notable decrease in accuracy was also evident in the specular reflection region, as shown in Figure 18d and Figure 19d.

**Figure 20:**
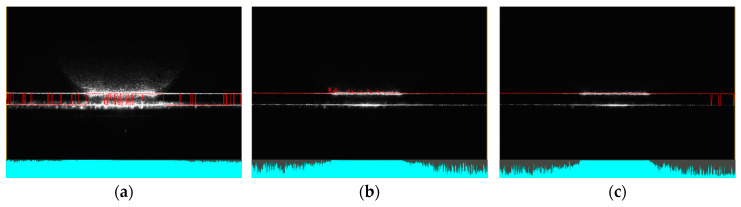
Sample of clear plastic, measured in the specular reflection area, roll of −20°and pitch of 0°. The image shows screenshots from the sensor controller. In the upper part, data from the camera are displayed, including reflected light and detected points connected by a red line. In the lower part, the reflected intensity of individual laser beams is shown (colored in cyan): (**a**) laser power of 100% and exposure time of 480 μs, standard detection; (**b**) laser power of 20% and exposure time of 480 μs, near detection; (**c**) laser power of 20% and exposure time of 240 μs, near detection.

In the case of transparent materials, light refraction could cause the detection of both top and bottom edges, as shown in Figure 20a. When scanning transparent materials, it is important to establish a mask or preferred reflection (either top or bottom), as seen in Figure 20b. The high laser power not only makes the entire profile visible but also causes significant overexposure of the image (Figure 20a). Reducing power helped partially eliminate overexposure. However, the main part of the image was still overexposed, resulting in an inaccurate profile, with step changes in the profile, as shown in Figure 20b. Smoothing the profile can be achieved by reducing the exposure time, as shown in Figure 20c. However, in the specular area, this may lead to reduced intensity at the boundary points of the profile and to the detection of points from the reflection of light from the bottom surface.

**Figure 21:**
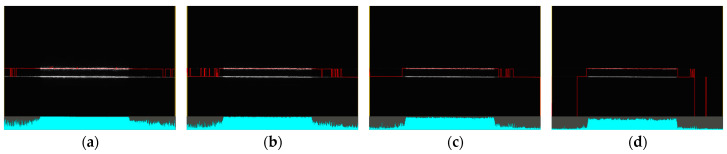
Sample of clear plastic, measured in the specular reflection area, roll of −17° and pitch of 0°, with varying exposure times. In the upper part, data from the camera are displayed, including reflected light and detected points connected by a red line. In the lower part, the reflected intensity of individual laser beams is shown (colored in cyan): (**a**) 240 μs; (**b**) 120 μs; (**c**) 60 μs; (**d**) 30 μs.

By adjusting the exposure time, it was possible to stabilise the central portion of the profile, as illustrated in Figure 21a–d. The figure shows data obtained from clear plastic in specular reflection (roll of −17° and pitch of 0°) with exposure times ranging from 240 μs to 30 μs. Trimming the profile by 20% on both sides helps to avoid capturing noise and allows for data collection with a stable profile.

For applications requiring accuracy within hundredths of a millimetre, it is crucial to take into account the warm-up time of the scanner to reach its operational temperature. Both manual and automatic parameter settings of the scanner (see Table 3) share a common limitation: when the sensor’s pitch angle exceeds 17° or is less than −17°, the number of detected points may remain consistent, but the profile shows increased noise and inaccuracies. Therefore, it is advisable to maintain the sensor’s orientation within the in-plane angle range of <−17°, 17°>, as larger tilt angles resulted in a significant deterioration of profile accuracy.

Automatic parameter adjustment is more suitable for scanning larger areas, such as when the scanner is mounted on an industrial robot and must operate across multiple angles. However, for scanning specific regions, manual parameter settings provide better precision and control. In manual scanning, the number of detected points can be increased by extending the exposure time, though this comes with the risk of increased noise; conversely, shorter exposure times reduce noise but may capture fewer points. For transparent materials, an exposure time of 120 μs was suitable in areas of specular reflections and 240 μs outside those areas, while 480 μs was optimal for non-transparent materials.

## 5. Conclusions

Triangulation laser sensors are used in industry for the precise measurement of distances and object positions. They operate on the principle of laser beam reflection and analysis of the reflected light. These sensors are used in quality control and automated manufacturing processes, increasing efficiency and reducing errors. However, it is essential to obtain reliable data from the sensor to achieve accurate results.

In this article, we focused on enhancing the reliability of data collection when scanning with an LLT sensor at in-plane and out-of-plane angles. The Keyence LLT sensor LJ-X8080 was used for measurements. The positioning device was designed to allow for the precise tilting of samples and data acquisition at various positions.

For non-transparent black and grey plastics, the results demonstrate that both manual and automatic sensor settings can achieve comparable completeness in point detection. However, the automatic settings provided better profile accuracy.

Across samples of aluminium with varying roughness, similar trends were observed in heat maps. Manual settings struggled with point detection in the specular reflection area. Automatic settings captured more points overall and provided better accuracy in these regions.

The scanning of transparent plastics posed unique challenges due to light refraction. For clear plastic, manual settings detected points only near the specular reflection zone, whereas automatic settings captured points across a broader area, though with limitations in extreme positions and regions of specular reflection. For red and orange transparent plastics, similar trends were observed, with manual settings providing high accuracy but limited coverage and automatic settings expanding the detection area while maintaining comparable accuracy.

Manual settings are suitable for tasks that require controlled conditions and high precision in limited regions, such as the specular reflection area. Automatic settings are advantageous for broader applications, offering greater adaptability to challenging surface properties and improving data reliability in less controlled environments.

While the proposed optimisation approach provides valuable insights, it also has certain limitations. First, it is highly dependent on the specific type and geometry of the LLT sensor used. Variations in sensor design, such as differences in triangulation base and camera angle, could lead to a different optimal positioning, requiring additional testing and adjustment. Second, the study is limited to a specific set of materials, and the sensor’s performance may differ significantly on surfaces with other optical or structural properties. Establishing a comprehensive digital database of results for various materials would be essential to generalising the approach. Third, practical deployment of the optimised sensor configuration may face geometric constraints in industrial environments, where physical obstructions or existing equipment can limit the feasible positioning of the sensor, leading to necessary compromises between accuracy and practicality.

Future research could delve into the detailed parameters required for scanning transparent materials and their relative positioning to the sensor. Furthermore, the scope of this methodology could be extended to semi-transparent materials. Another area for potential research is the dynamic optimisation of sensor settings and parameters, such as laser power and exposure time, using feedback from the sensor. These findings could also be applied to digital twins of sensors [35,36] or even entire technological processes [37].

## Figures and Tables

**Table 1:** Parameters of the LJ-X8080 sensor [33].

Parameter	Specification
Reference distance (*Z*-axis)	73 mm
Measuring range (*Z*-axis)	±20.5 mm (full scale = 41 mm)
Measuring range (*X*-axis)	30 mm (near side)
	35 mm (reference distance)
	39 (far side)
Linearity (*Z*-axis)	±0.03% of the full scale
Profile data count	3200 points
Laser type	Blue laser
Laser source	10 mW
Laser wavelength	405 nm (visible light)

**Table 2:** Known parameters of scanned objects.

Material	Width [mm]	Height [mm]	Thickness [mm]	Roughness
Non-transparent plastics	100	100	3	Ra 1.2
Coloured transparent plastics	100	100	3	Ra 0.01–0.04
Pure transparent plastic	100	100	5	Ra 0.01–0.04
Aluminium alloy	40	80	12	Ra 0.8
				Ra 1.6
				Ra 3.2
				Ra 6.3
				Ra 12.5

**Table 3:** Description of sensor configuration parameters [33].

Parameter	Description	Value
Dynamic range	It specifies the light-receiving sensitivity range of the capture element in the sensor unit. For high precision, the dynamic range is lowered, and the peak is measured at high sensitivity. This is used for target objects that have a small difference in reflectance.	1 to 9
Exposure time	It sets the exposure time of the capture element in the sensor unit. It is the length of time in which the camera collects light from the sample.	15 μs, 30 μs, 60 μs, 120 μs, 240 μs, 480 μs, 960 μs, 1700 μs, 9.6 ms
Detection sensitivity	It sets the threshold value for the received light quantity to be detected. Increasing this value makes it easier for a received light quantity to be detected. Reduce this value to prevent mis-detection due to ambient light or multiple reflected lights.	1 to 5
Laser power	Laser power in percentage. The maximum power is 10 mW.	1–100%

**Table 4:** Specific parameter values for manual and automatic sensor settings.

Parameter	Manual Settings	Automatic Settings		
		Aluminium Alloys and Grey Plastic	Black Plastic	Transparent Plastics
Dynamic range	1	7	9	1
Exposure time	120 μs (transparent, roll, and pitch) 240 μs (transparent and roll) 480 μs (other materials)	480 μs	9.6 ms	9.6 ms
Detection sensitivity	3	3	3	3
Laser power	100 (black plastic) 40 (aluminium alloys) 20 (grey and transparent plastics)	1–100 (auto)	1–100 (auto)	1–100 (auto)

## Data Availability

Contact the corresponding author.
